# Prevalence and severity of gingivitis in school students aged 6–11 years in Tafelah Governorate, South Jordan: results of the survey executed by National Woman’s Health Care Center

**DOI:** 10.1186/s13104-015-1532-y

**Published:** 2015-11-09

**Authors:** Rania Rodan, Feryal Khlaifat, Leena Smadi, Reem Azab, Asma Abdalmohdi

**Affiliations:** Royal Medical Services, P.O Box 391, Salt, 19110 Jordan; Ministry of Health, P.O Box 1040, Amman, 11941 Jordan; Department of Conservative Dentistry, University of Jordan, P.O Box 855066, Amman, 11855 Jordan; Ministry of Health, P.O Box 86, Amman, 11118 Jordan; Head of Planning, Research and Information Department, National Woman’s Health Care Center, P.O Box 723, Amman, 11831 Jordan

**Keywords:** Gingivitis, Prevalence, Oral hygiene, Gingival index, Plaque index

## Abstract

**Background:**

A cross-sectional census was conducted on 994 public school students aged 6-11 years living in 3 different parts of Tafeleh Governorate—South of Jordan, to determine the prevalence, and severity of gingivitis and to evaluate the oral hygiene habits among them as a part a survey executed by National Woman’s Health Care Center. All students were examined for gingival index (GI) and plaque index (PI), information about oral hygiene habits was recorded.

**Results:**

Only 29.8 % had healthy gingiva, 38.5 % had mild gingivitis, 31.4 % had moderate gingivitis, and 0.3 % had severe gingivitis. The difference between both genders was not statistically significant P > 0.05. 36.8 % of the examined students never brushed their teeth. Average gingival index (GI) and average plaque index (PI) were 0.77 and 0.61 respectively.

**Conclusions:**

Fair oral hygiene with mild to moderate gingivitis is highly prevalent among Tafelah school children. This study indicated that oral health status among schoolchildren in Tafelah is poor and needs to be improved. Long-term school based oral health education programme is highly recommended.

**Electronic supplementary material:**

The online version of this article (doi:10.1186/s13104-015-1532-y) contains supplementary material, which is available to authorized users.

## Background

Periodontal diseases are the ones of the most prevalent oral diseases that get its roots early in childhood [1, 2]. As a consequence of these diseases, if they are not treated on time, the destructive processes are progress in both solid and soft tissues together with losing teeth [3].

Adequate daily removal of dental plaque prevents periodontal diseases and dental caries [3]. The most common and effective way to promote oral hygiene is tooth-brushing; therefore brushing is recommended to be adopted as a habit, which is repeated every morning and evening, at least twice a day. In addition to improved oral hygiene, which prevents periodontal diseases, frequent brushing with fluoride toothpaste increases the resistance of dentition to dental caries [4, 5].

Tooth brushing and other behaviors that comprise young people’s lifestyles may directly or indirectly impinge on their health in the short or long term. Most of behavioral patterns were established in early childhood. Oral health behavior may constitute an integral part of an individual’s lifestyle. It is essential to develop an effective education programs for oral health and practice targeted at young people.

Periodontal diseases including gingivitis and periodontitis are serious infection that if left untreated can lead to tooth loss. Gingivitis is reversible with professional treatment and good oral home care, whereas periodontitis is irreversible as this progress with destruction of bone. Untreated gingivitis can advance to periodontitis. Hence if gingivitis and periodontitis are assessed in an early stage it will minimize the chance of tooth loss. Epidemiological studies are helpful in planning and implementing oral health programs. This would help in combating these diseases.

According to the World Health Organization (WHO), oral health is integral to general health and essential for well-being [6]. Surveillance of oral health on community level thus has to be done at regular intervals.

One of the tasks of the Jordanian National Woman’s Health Care Center is to assess the health status and needs of the underprivileged communities throughout Jordan by collection and interpretation of reliable health information.

**Fig. 1:**
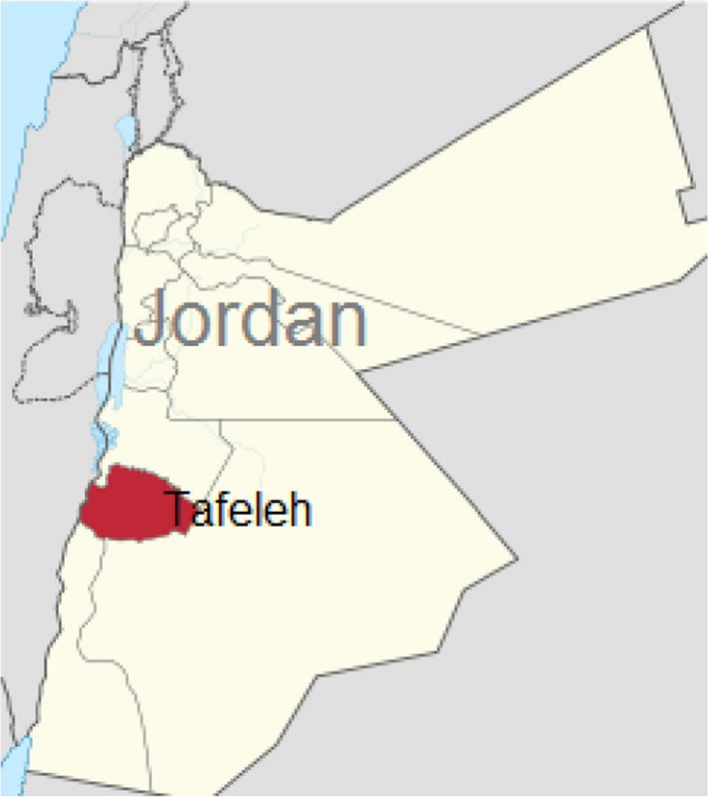
Tafelah Province: this map shows the location of Tafelah province within Jordan, it shows also the relative size of this province to the total size of Jordan. Source https://upload.wikimedia.org/wikipedia/commons/2/25/Tafilah_in_Jordan.svg

Tafelah is one of these communities, (Fig. 1) it is a governorate in south of Jordan with a population estimated by the Department of Statistics of Jordan at the end of year 2012 to be 89,400, which account for 1.4 % of Jordan Population (of them 45,500 males and 43,900 females). Tafelah is divided into three districts: Tafelah District (62.3 % of population), Bsaira District (25.7 %) and Hasa District (12 %).

The number of schools in Tafelah is 48, 41 of them are for boys and rest are for girls accommodate 26,548 students (13,085 girls and 13,463 boys). The population of Tafelah is served by 16 dental clinic distributed throughout the governorate [7].

The Oral Health Survey (OHS) 2014 for students aged 6–11 years of age was conducted at Tafelah Governorate schools (south of Jordan) to obtain relevant information on both the oral health condition of the students in this underprivileged area of Jordan and to assess the oral health related behaviors of the students in this area. The survey focuses on two most common but preventable oral diseases, tooth decay (dental caries) [8] and gum disease (periodontal disease), which affect many students; in this article we will review the results of gum disease part of the survey.

## Methods

The survey methodology comprised of a series of fieldwork surveys which were conducted from October 2013 through March 2014. The survey includes students aged 6–11 years of age in six public schools within the governorate (distributed between all districts).The schools selection was based on the two largest schools per district, these schools include classes with mixed gender (grade 1–grade 4) after which mixed classes are not allowed and male students have to move to a boys only schools, all students examined after grade 4 were girls (age group 10–11).

The study protocol was approved by the ethical committee at Ministry Of Health. Due to many logistic barriers it was difficult to get written parents’ permission for each examined student individually, though all parents were informed by a letter sent with their children informing them of the screening and examination which will be done as part of Ministry of Health, Ministry of Education and the National Women Health Care cooperation programme. We did not have any single refusal or objections from students’ parents; the ethical committee was satisfied with this arrangement.

The clinical examination was carried out by three dentists (examiners) all through the survey. The clinical judgment differences were minimized through pre- survey training for all examiners to homogenize and standardize the examination. Information on the behavior of students was collected using a questionnaire which was completed by the students themselves with the assistance of specially trained dental nurse. Before the survey, the draft questionnaire was pre-evaluated by dentists and dental care nurses working for the School Dental Care Service of the Ministry of Health. Several revisions were made on the questionnaire before it was finalized. Students were examined in a mobile dental clinic which is fully equipped with all the necessary examination instruments, cross infection control methods.

In the process of the research, all the schoolchildren were interviewed according to a standard questionnaire (Additional file 1). Clinical examinations were carried out according to World Health Organization (WHO) methods [9]. A mouth mirror and a WHO ball-tip probe were used for the examination. Oral plaque was estimated by running the side of the probe along the teeth surfaces. The children were examined for oral hygiene status, gingival conditions and dental caries. Oral hygiene was evaluated by examining the dental plaque present on the inner and outer aspects of the six index teeth, using the criteria of the plaque index of Silness and Löe [10]. The six indexed teeth are: upper right first molar, upper right lateral incisor, upper left first premolar, the lower right first premolar, the lower left lateral incisor and first molar. Missing teeth are not substituted. The gingival condition was determined for the same teeth using the criteria of the gingival index of Löe and Silness [11].

Table 1 shows the criteria used for plaque index according to Silness-Löe.

**Table 1:** Criteria used for plaque index (PI)

Score	Criteria
0	No plaque
1	A film of plaque adhering to the free gingival margin and adjacent area of the tooth. The plaque may be seen in situ only after application of disclosing solution or by using the probe on the tooth surface
2	Moderate accumulation of soft deposit s within the gingival pocket, or the tooth and gingival margin which can be seen with the naked eye
3	Abundance of soft matter within the gingival pocket and/or on the tooth and gingival margin

Objective research was performed evaluating the state of oral hygiene during the examination as follows: Good (plaque index 0.0 i.e. absence of plaque), Fair (plaque index 0.1–1.9 i.e. presence of plaque) Bad (plaque index 2.0–3.0 i.e. plaque seen by naked eye) [12].

The gingival index, given by Löe and Silness [9] was used for recording the severity of gingivitis. Gingival index, given by Loe and Silness 1963 measures severity of gingivitis on a scale ranging from 0.1 to 3.0 (0.1–1.0: mild gingivitis, 1.1–2.0: moderate gingivitis, and 2.1–3.0: severe gingivitis) [13, 14].

Table 2 shows the criteria used for gingival index according to Löe and Silness 1963.

**Table 2:** Criteria used for gingival index (GI)

Score	Criteria
0	No inflammation
1	Mild inflammation, slight change in color, slight edema, no bleeding on probing
2	Moderate inflammation, moderate glazing, redness, bleeding on probing
3	Severe inflammation, marked redness and hypertrophy, ulceration, tendency to spontaneous bleeding

*Statistical analysis* The analysis of the data was performed using SPSS 20.0 for Windows (SPSS Inc., Chicago, IL, USA).

## Results

### Demographic data

The sample population was 995 students, 64 % of them were females distributed almost equally over the three districts, and the students were examined in the 6 schools of the three districts.

### Oral hygiene behavior and status

**Fig. 2:**
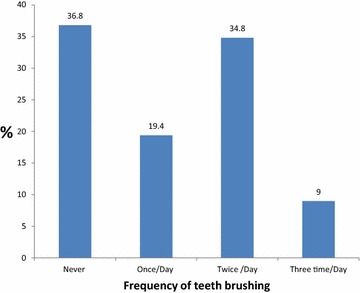
Frequency of teeth brushing. The frequency of teeth brushing as reported by students extracted from their answer using a pre- prepared questionnaire

**Fig. 3:**
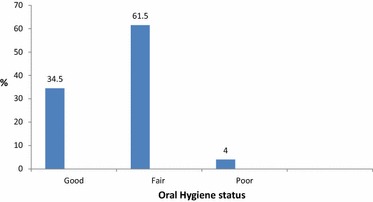
Oral hygiene status according to plaque index (PI). The oral hygiene status is demonstrated using the plaque index (PI) for all students examined during the survey, the status was classified as Good, Fair and Poor

Figure 2 shows that 36.8 % of the interviewed students stated that they never brushed their teeth, 19.4 % brush their teeth once per day, 34.8 % twice daily, 9.0 % three times a day. According to plaque index 34.5 % was considered to have good oral hygiene (plaque index 0.0), 61.5 % was considered to have Fair oral hygiene (plaque index 0.1-1.9) and 4.0 % was considered to have bad oral hygiene (plaque index 2.0–3.0). (Figure 3) The mean plaque index was 0.61 ± 0.57 for the whole sample, the difference between both genders was not statistically significant.

### Oral health status

**Fig. 4:**
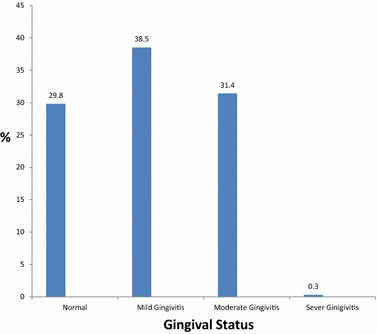
Gingival status according to gingival index (GI). The Gingival status is demonstrated using the gingival index (GI) for all students examined during the survey, the status was classified as Normal, Mild Gingivitis, Moderate Gingivitis, and Sever Gingivitis

Among the students examined 70.2 % had gingivitis. According to gingival index 29.8 % had healthy gingiva, 38.5 % had mild gingivitis (GI 0.1–1.0), 31.4 % had moderate gingivitis (GI 1.1–2.0), and 0.3 % had severe gingivitis (GI 2.1–3.0). (Figure 4) The mean gingival index was 0.77 ± 0.68 for both genders.

## Discussion and conclusions

In Jordan very few studies looked at oral health among school children, all of them neither examined oral health at neither south of Jordan nor studied specifically prevalence of gingivitis in primary schools [15–18].

The present study is among the foremost efforts to determine the oral behaviors and prevalence and severity of gingivitis in school children aged 6–11 in one of the most unprivileged areas of Jordan.

This survey was also conducted to assess the need to start a local campaign to raise the public awareness for oral hygiene and the need to modify polices for early access to preventive dental services among school children.

The results of this surveys shows that 69.9 % of school children aged 6-11 had gingivitis of mild to moderate severity. Those results are similar to the results of other parts of the word of similar cultural and socioeconomic status. A recent survey in Lucknow/India of school children aged 8–16 have shown that 71.11 % of school children aged 8–10 suffered of gingivitis of mostly mild to moderate severity [13]. Another survey on Lithuanian school children of older age group of 11–15 have also showed that >50 % of them complaining of gum bleeding [19]. In Kaunas a survey have shown that 59.6 % of school children aged 6–8 suffered of light gingivitis [20]. In Tehran it was shown that 87.7 % of schoolchildren aged 9–13 had gingivitis [21]. On the other hand a survey on periodontal status of Greek 12 year’s old population has shown that 41.5 % had bleeding on probing [22]. Gingival bleeding was found in 32.8 % of the children aged 6–12 years from the Danube Delta Biosphere Reserve [23].

Gingival index and plaque index were chosen for this study as it has been widely used to evaluate the level gingival inflammation and the level of oral cleanliness in epidemiological studies [14]. This relatively simple assessment is fairly reproducible, easy to use since the criteria are objective and the examinations can be carried out quickly with a high level of reproducibility and with minimum training.

This study revealed that the mean plaque index and gingival index for the total subjects was 0.61 and 0.77 respectively. Those results were interpreted that school children of this age group 6–11 had fair oral hygiene with mild gingivitis. Regarding gender variations, the difference was statistically non-significant, (*p* = 0.636 for PI and 0.790 for GI) between both genders. These findings disagreed with earlier studies in 13–14 year old Northern Jordanian schoolchildren that reported plaque index scores were 1.82 and 1.63 during 1993 and 1999 respectively [24]. Furthermore, gingival index scores reported in the same study [24] were 1.89 in 1993 and 1.67 in 1999 which were also higher than the figure found in this study. A more recent study took place in 2006 for 14–15 year old schoolchildren in Jerash district and reported a plaque index score of 1.46 and gingival index score of 1.56 [15]. But these results are still higher than results obtained in this study. The higher results can be explained by the difference in the age group, a previous cross-sectional study of gingival health status among 5- and 12-year-old children in Yemen showed increased prevalence and severity of gingivitis in a 12 years old children when compared to a 5 years old children as the plaque index increased as well [14]. Regarding gender variations, a previous study that described trends in oral health in Jordanian male and female schoolchildren, showed that boys had higher plaque and gingival scores than girls within this age group. This gender difference with regard to plaque and gingival scores may be related to the patterns of personal oral hygiene, hormonal changes occurring during puberty and grooming effect at this age [24]. Another study found that girls scored more favorably on behavioral measures, showed more interest in oral health, and perceived their own oral health to be good to a higher degree than did boys [19]. Those gender differences were not obvious among school children surveyed in this study.

With regard to hormonal changes occurring during puberty that affect the gingiva, several cross-sectional studies have demonstrated an increase in gingival inflammation without an accompanying increase in plaque levels during puberty [25].

Tooth brushing is not common or routinely practiced in schoolchildren in Tafelah. The proportion of children reported to be brushing at least once to twice per day was 54.2 % in the Tafelah health survey. Although there is good evidence to recommend tooth brushing twice daily for control of plaque and gingivitis [5], 36.8 % of 6–11 year-old children reported that they never brushed their teeth. Routine dental visits are not a habit for the children in Tafelah. Results showed that 85.9 % of the surveyed school children had never been to a dentist.

Unlike the results of this survey where frequency of teeth brushing was similar in boys and girls several other epidemiological surveys have shown that the factor most consistently associated with teeth brushing and other oral hygiene habits frequency was gender. Girls were more concerned about their personal hygiene than boys.

They have also shown the importance of social-economic background for determining children’s tooth brushing behavior [26]. Children from rural areas reported higher percentage of inadequate oral hygiene than children from urban areas [19]. Similar results were also demonstrated in public schools when compared to private schools [27] and in lower socio-economic background when compared to a higher socio- economic background [26]. As all the surveyed students were from public schools and almost of similar socio- economic background this factor was not investigated in our survey.

Oral health education is an important aspect. All students should get at least one oral health education lesson with a supervised tooth brushing exercise every school year. Oral health education sessions should be organized for parents and pregnant mothers and dental health education programmes should be conducted for schoolteachers.

Post-intervention 9 months oral health education on oral hygiene knowledge, practices, plaque control and gingival health of 13- to 15-year-old school children in Bangalore city, there was significant improvement in oral hygiene knowledge and practices in experimental groups. There were significant reductions in mean plaque index and gingival index scores in the experimental groups [28]. Plaque and Gingival score reductions were highly significant after a school dental education program in improving oral health knowledge and oral hygiene practices and status of 12- to 13-year-old school children, and were not influenced by the socioeconomic status. When oral health knowledge was evaluated, highly significant changes were seen in intervention schools; more significantly in schools receiving more frequent interventions [29]. A cluster randomized controlled trial education programme for 6-year-olds concluded that oral hygiene instruction to 6-year-old children and their parents improves their dental health [30].

Prevention and treatment of early stages of periodontal diseases are relatively simple and very effective. In many cases it is enough to have the social education and instructions for correct and regular oral hygiene. The preventive component of lifestyle should be induced and accepted at a young age. Epidemiological investigations of periodontal status and oral hygiene are an important part of such preventive program.

- Information provided by the present study can be used as preliminary data and further large scale epidemiological studies can be under taken to assess and confirm other dental diseases and their associated risk factors in Tafelah, Jordan. School dental health programs and dental camps at school level are necessary in this region and they should be conducted at regular intervals, because children in this region do not have accessed to standard dental care and treatment. Because schools children do not know much about dental diseases, methods of their prevention and to maintain proper oral hygiene, therefore education and motivation of children is of paramount important in this region. Teachers and parents should be taught and encourage to inculcate healthy life style habits in children. However education itself is not enough to bring tangible changes in behavior change. Another opportunity to utilize the school setting would be to ensure lifelong skills such as school based tooth brushing and hand washing. It is worth mentioning that we utilize this survey as an opportunity to organize oral health education lessons for the surveyed school children and their school teachers to increases dental awareness and to enhance oral hygiene methods by demonstrating the correct frequency, method and duration of teeth brushing. Intersectoral coordination with education, government sectors and development of public health policy has profound effect in improving the health of the community people.

## Additional file

10.1186/s13104-015-1532-y Questionnaire answered by students and examinatiuon carried out by dentists.

## Abbreviations

GI: gingival index
PI: plaque index
WHO: World Health Organization (WHO)
OHS: oral health survey (ohs)
