# Supplementary material for: Prevalence and severity of gingivitis in school students aged 6–11 years in Tafelah Governorate, South Jordan: results of the survey executed by National Woman’s Health Care Center
Source: BMC Res Notes. 2015 Nov 9;8:662. doi: 10.1186/s13104-015-1532-y (PMC4640206; doi:10.1186/s13104-015-1532-y)
Supplement: Supplementary file 1 — 10.1186/s13104-015-1532-y Questionnaire answered by students and examinatiuon carried out by dentists. [file 13104_2015_1532_MOESM1_ESM.docx]

| المدرسة: | اسم الطالب: |
| --- | --- |
| الصف: | **تاريخ الميلاد:** |
| Medical history: |  |
| Dental history: |  |
| Oral hygiene measures: | **Frequency:** |
| Extra oral examination: |  |
| - Facial symmetry |  |
| - Lymph nodes |  |
| - TMJ |  |
| Intra oral examination: |  |
| Soft tissue |  |
| - Oral hygiene |  |
| - Gingiva |  |
| - Mucosa |  |
| - Tongue |  |
| Gingival index (0-3)  0=Normal gingiva  1=Redness  2=Bleeding on probing(BOP)  3=Profuse spontaneous bleeding | **Plaque index (0-3)**  **0=No plaque**  **1=Plaque not seen but detected by probe**  **2=Obvious plaque < 1/2 crown**  **3=Obvious plaque>2/3 crown** |
| \|  \|  \|  \| \| --- \| --- \| --- \| \| **6** \| **2** \| **4** \| \| **4** \| **2** \| **6** \| \|  \|  \|  \| | \|  \|  \|  \| \| --- \| --- \| --- \| \| **6** \| **2** \| **4** \| \| **4** \| **2** \| **6** \| \|  \|  \|  \| |
| Hard tissue |  |
| Deciduous teeth (DMF)  D= Decay M=Missing F= Filling | **Permanent teeth (DMF)** |
| \|  \|  \|  \|  \|  \|  \|  \|  \|  \|  \| \| --- \| --- \| --- \| --- \| --- \| --- \| --- \| --- \| --- \| --- \| \| **E** \| **D** \| **C** \| **B** \| **A** \| **A** \| **B** \| **C** \| **D** \| **E** \| \| **E** \| **D** \| **C** \| **B** \| **A** \| **A** \| **B** \| **C** \| **D** \| **E** \| \|  \|  \|  \|  \|  \|  \|  \|  \|  \|  \| | \|  \|  \|  \|  \|  \|  \|  \|  \|  \|  \|  \|  \|  \|  \|  \|  \| \| --- \| --- \| --- \| --- \| --- \| --- \| --- \| --- \| --- \| --- \| --- \| --- \| --- \| --- \| --- \| --- \| \| **8** \| **7** \| **6** \| **5** \| **4** \| **3** \| **2** \| **1** \| **1** \| **2** \| **3** \| **4** \| **5** \| **6** \| **7** \| **8** \| \| **8** \| **7** \| **6** \| **5** \| **4** \| **3** \| **2** \| **1** \| **1** \| **2** \| **3** \| **4** \| **5** \| **6** \| **7** \| **8** \| \|  \|  \|  \|  \|  \|  \|  \|  \|  \|  \|  \|  \|  \|  \|  \|  \| |
| Diagnosis: |  |
| Recommendations: |  |
| Doctor name: | **Signature:** |
